# Limited discriminatory value of RAMRIS findings between RA and hand osteoarthritis: a cross-sectional MRI study

**DOI:** 10.1007/s00296-026-06085-5

**Published:** 2026-02-14

**Authors:** Philipp Sewerin, David Kiefer, Jean Maurice Arcq, Styliani Tsiami, Johanna Mucke, Anna Kernder, Uta Kiltz, Xenofon Baraliakos

**Affiliations:** 1 Ruhr-Universität Bochum, Bochum, Germany; 2 Ruhr-Universität Bochum, Rheumazentrum Ruhrgebiet, Claudiusstrasse 45, 44649 Herne, Germany

**Keywords:** Rheumatoid arthritis (RA), OA, Imaging-MRI

## Abstract

To evaluate the capacity of the RA MRI Score (RAMRIS) in distinguishing rheumatoid arthritis (RA) and hand osteoarthritis (HOA). Patients with RA and HOA were retrospectively matched in pairs. Inflammatory and degenerative changes in MRI scans of the hands were quantified and compared using RAMRIS, focusing on bone marrow edema (BME), erosion, synovitis, and tenosynovitis. CRP, ESR, and autoantibodies (RF and ACPA) and clinical scores (DAS-28, FFbH, and pain level), smoking status, and medication use were assessed additionally. Overall, 100 RA and 100 HOA patients with confirmed diagnosis were included. Age, pain severity, and functional impairment did not differ between groups. RAMRIS revealed significantly higher synovitis subscores in RA compared to HOA (*p*<0.001), while no significant differences were found for BME (*p* = 0.076), erosion (*p* = 0.366), or tenosynovitis (*p* = 0.129). Higher RAMRIS scores were observed at the level of individual joints in RA. The mean erosion-subscore was found to be higher in RA males than in RA females, though this was not observed in all joints. In HOA, men exhibited a mean erosion score that was higher than that of women. Smoking status had limited association with RAMRIS findings, but RA non-smokers exhibited greater inflammatory burden than HOA non-smokers in multiple joints. RA exhibited significantly higher synovitis subscores, indicative of active inflammation and leading to overall higher RAMRIS scores, compared to HOA. However, HOA demonstrated high RAMRIS scores for synovitis, tenosynovitis, erosions, and BME. MRI may assist in distinguishing RA from HOA; however, its interpretation must be integrated into the clinical context.

## Introduction

Rheumatoid arthritis (RA) is one of the most prevalent inflammatory rheumatic diseases, affecting up to 1% of the population [1]. It is characterized by typically symmetrical polyarthritis of the small joints in the hands and feet. If left untreated, RA can lead to progressive, irreversible erosive changes that may result in functional impairment over time. Early diagnosis is crucial for effective disease control and damage prevention [2]. To facilitate early detection, imaging techniques such as ultrasonography and magnetic resonance imaging (MRI) are increasingly being used in both clinical trials and routine practice [3]. Unlike conventional radiography, which can only identify structural damage after it has occurred, MRI enables the detection of early-stage and even subclinical lesions due to its superior spatial resolution [4].

However, MRI findings must be carefully interpreted, as small lesions in bone and soft tissues can also be observed in healthy individuals [5]. Due to a lack of reference data, such changes may be misclassified as pathological when they may simply reflect mechanical stress or coexisting osteoarthritis (OA). Inflammatory findings such as synovitis, tenosynovitis, and bone marrow edema (BME) are strongly associated with active RA but can also appear in other conditions [4, 6].

Apart from RA, degenerative joint diseases leading to polyarthritis of the fingers are common, affecting up to 15% of the general population, with a higher prevalence in women [7–9]. In contrast to RA, hand osteoarthritis (HOA) primarily affects the proximal interphalangeal (PIP) and distal interphalangeal (DIP) joints, as well as the first carpometacarpal (CMC-1) joint (rhizarthrosis), whereas the metacarpophalangeal (MCP) joints are less frequently involved. Palpable osteophytes are a hallmark of HOA [8, 10, 11]. Additionally, episodes of activated OA may present with local synovitis, resembling inflammatory arthritis [12]. A large ultrasonography study has demonstrated that osteophytes are present in nearly all individuals over 50, even in the absence of clinical OA. However, valid data on the prevalence and distribution of MRI-detected synovitis, tenosynovitis, and BME in HOA remain scarce [7].

MRI is widely used in both research and clinical practice due to its high sensitivity in detecting changes in bones, soft tissues, tendons, ligaments, and entheses. Numerous studies have demonstrated its ability to detect early, and even subclinical, lesions in RA patients [4, 5]. Notably, MRI is the only imaging modality capable of identifying BME, a key predictor of future erosive changes in RA. MRI can also aid in distinguishing between various inflammatory arthritis; for instance, synovitis and tenosynovitis are more frequent in RA than in psoriatic arthritis (PsA), whereas periosteal involvement is more commonly observed in PsA [13].

Despite the growing use of MRI in rheumatology, data on HOA remain limited, and large-scale MRI studies assessing specific HOA-related findings using standardized scoring systems, such as the RA MRI Score (RAMRIS), are lacking.

This study aimed to evaluate the capacity of RAMRIS in distinguishing RA from HOA in MRI examinations from routine clinical practice and to correlate imaging findings with clinical and demographic patient data.

## Materials and methods

### Study population

Patients with confirmed diagnosis of clinically active rheumatoid arthritis (RA) according to EULAR classification criteria for RA [14] were consecutively selected from the hospital’s information system starting in 2022 based on the confirmed diagnosis of RA and the availability of complete hand MRI scans. These consecutive patients were 1:1 matched by age and sex with patients diagnosed with hand osteoarthritis (HOA) according to EULAR classification criteria for HOA confirmed by conventional x-rays [9] who also had complete MRI scans.

Clinical and demographic data were collected for all participants, including erythrocyte sedimentation rate (ESR, normal < 20 mm/h), C-reactive protein (CRP, (normal < 0.5 mg/dl)), rheumatoid factor (RF, (normal < 14 IU/ml)), anti-citrullinated protein antibodies (ACPA), pain intensity using the Numerical Rating Scale (NRS), functional status assessed by the Hannover Functional Questionnaire (Funktions-Fragebogen Hannover, FFbH), and disease activity measured with the Disease Activity Score 28 (DAS-28 ESR). Additionally, data on pharmacological treatments, such as glucocorticoids (GCs), nonsteroidal anti-inflammatory drugs (NSAIDs), conventional synthetic (cs) and biological (b) disease-modifying antirheumatic drugs (DMARDs), and analgesics were recorded. Smoking status was also documented. An overview of patient characteristics is provided in Table 1.

**Table 1:** Demographics and clinical parameters of patients with hand osteoarthritis (HOA) and rheumatoid arthritis (RA)

Demographics	HOA (*n* = 100)			RA (*n* = 100)	
	Mean	SD		Mean	SD
Age, in years	52.3	7.15		52.6	7.10
pain scale, NRS 0–10	7.3	2.47		7.3	2.45
FFbH	71.1	22.76		66.9	24.75
DAS-28 ESR	3.9	1.44		4.9	1.13
C-reactive protein	1.2	4.30		1.8	3.01
Rheumatoid factor	17.0	41.62		63.1	95.75
ACPA	12.8	40.84		84.2	107.40
ESR	13.6	17.42		22.2	21.26
Mean glucocorticoid dosis (mg/day)	2.5	7.45		4.2	7.88

CPA anti-citrullinated protein antibody, cs DMARD = conventional synthethic disease- modifying antirheumatic drugs, NSAIDs = nonsteroidal anti-inflammatory drugs, bDMARDs biological disease- modifying antirheumatic drugs

## MRI acquisition and image analysis

In accordance with the MR scanning protocols recommended by the European Society of Musculoskeletal Radiology Arthritis Subcommittee for the assessment of inflammatory changes of the musculoskeletal system in the course of rheumatic diseases [15] and the availability and capacity of the MRI scanners at the radiology department in Herne, the following four MRI sequences with field strengths (fs) of 1,5 Tesla (T) were used for the examinations:

1. T1-weighted spin-echo, transverse view (T1_se_tra; slice thickness: 3 mm).
2. T1-weighted turbo spin-echo, coronal view (T1_tse_cor; slice thickness: 2.5 mm).
3. Turbo inversion recovery magnitude, transverse view (T2_tirm_tra; slice thickness: 3 mm).
4. Proton density-weighted turbo spin-echo with fat suppression (Dixon technique), coronal view (Pd_tse_fs_dixon; slice thickness: 2.5 mm).

All MRI scans were acquired using the same 1.5T scanner, with a slice thickness of 3 mm and an interslice gap of 0.3 mm, as used in daily practice, while the most symptomatic hand was examined Synovitis was scored according to Frenken et al. using proton density-weighted turbo spin-echo with fat suppression, as contrast agents were not used [16].

Following patient selection, all MRI images were anonymized by an independent investigator who was blinded to clinical data. Image evaluation was conducted by a in a two-rater consensus-read by two experienced readers using the RA MRI Score (RAMRIS) on the Radiant DICOM viewer 5.5.1 (64-bit).

### RAMRIS scoring

The Rheumatoid Arthritis Magnetic Resonance Imaging Score (RAMRIS), developed by the international rheumatology network OMERACT, was used as the scoring system in this study. Between 1998 and 2002, the OMERACT MRI Working Group developed and validated the OMERACT 2002 RAMRIS, providing a standardized approach for RA joint evaluation that is widely applicable across rheumatology centers [17]. Synovialitis was scored according to RAMRIS-SAFE using proton density turbo spin echo fat suppression Dixon technique (Pd_tse_fs_dixon, slice thickness 2.5 mm) [16].

Although RAMRIS was originally developed for RA, this study also assessed its applicability in scoring hand osteoarthritis (HOA) and comparing its diagnostic utility between RA and HOA.

### Statistical analysis

All statistical analyses were performed using SPSS software, version 26.0. Descriptive statistics, including mean values, standard deviations, and frequencies, were calculated. Comparisons between groups were conducted using an independent t-test for continuous variables and a chi-square test for categorical variables. A p-value < 0.05 was considered statistically significant.

The study was conducted in accordance with the Declaration of Helsinki and was approved by the institutional ethics committee of the Ruhr-University of Bochum (approval number 2016-379-f-S).

## Results

A total of 200 patients were included, *n* = 100 with confirmed RA and *n* = 100 with confirmed HOA. Both groups consisted of *n* = 50 male and *n* = 50 female patients, respectively. The mean age of patients was 52.6 ± 7.10 years in the RA group and 52.3 ± 7.15 years in the HOA group.

Patients with RA exhibited significantly higher DAS-28-ESR, CRP, ESR, RF, and ACPA values compared to those with HOA. Additionally, bDMARDs were only used in RA patients. Therapies are listed in Table 2.

**Table 2:** The proportion of patients with elevated serum levels of serological markers, drug treatment and lifestyle of patients with hand osteoarthritis (HOA) and rheumatoid arthritis (RA)

Proportion of patients (%)	HOA (*n* = 100)	RA (*n* = 100)
Elevated C-reactive protein	10	55
Rheumatoid factor	6	47
ACPA	5	46
csDMARDs	8	21
NSAIDs	53	57
Glucocorticoids	16	33
bDMARDs	0	7
Analgesics	25	21
Smoking	53	50

In contrast, NRS pain scale scores, analgesic use, and smoking habits were similarly distributed between the two groups (Tables 1 and 2).

## Imaging results

The mean RAMRIS score was vs. 13.71 for RA and 10.92 for HOA (*p* < 0.001). A comparison of total RAMRIS subscores for erosions, bone marrow edema (BME), synovitis, and tenosynovitisacross the entire study cohort revealed that synovitis subscores were significantly higher in RA patients compared to HOA patients (*p* < 0.001) (Table 3). While synovitis was notably more prevalent in RA, the differences in erosion, BME, and tenosynovitis scores were numerically but not statistically significant different (Table 4).

**Table 3:** MRI sum of RAMRIS-subscores of patients with hand osteoarthritis (HOA) and rheumatoid arthritis (RA)

Mean RAMRIS scores	HOA (*n* = 100)			RA (*n* = 100)			
	Mean	SD		Mean	SD		*p*-value
erosion	5.1	4.75		6.5	7.87		0.366
bone marrow edema	1.4	2.42		5.0	9.75		0.076
synovitis	4.6	2.84		6.5	3.98		0.001
tenosynovitis	1.2	0.80		1.4	0.74		0.129

**Table 4:**
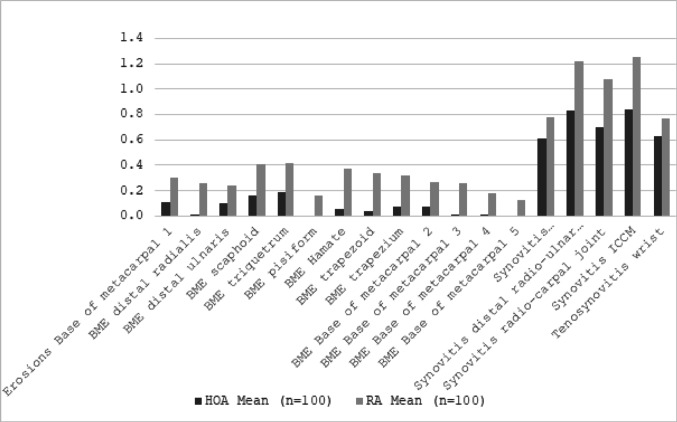
MRI mean RAMRIS-subscores of individual joints and bones of patients with hand osteoarthritis (HOA) and rheumatoid arthritis (RA)

BME = bone marrow edema, ICCM = Intercarpal carpometacarpal joint = carpometacarpal joint

A more detailed analysis of individual RAMRIS regions revealed that erosions were significantly more frequent at the base of MCP 1 in RA patients compared to those with HOA. Bone marrow edema (BME) was observed more commonly across nearly all carpal bones in RA patients. While BME was also present in HOA patients, it occurred less frequently and did not reach statistical significance.

Differences in BME subscores were particularly pronounced at the MCP joints, with no BME detected at the base of MCP 4 and 6 in HOA patients. In terms of synovitis, RA patients exhibited a significantly higher prevalence of synovial changes in the carpus compared to HOA patients, further reinforcing the inflammatory nature of RA-related joint involvement.

In the tables below (Tables 5 and 6) only statistically significant (*p* < 0.005) results are shown. Specific RAMRIS-subscores of erosions, BME, synovitis and tenosynovitis were more common in male HOA patients than in female HOA patients (erosions in the distal radius, base of metacarpal 3; bone marrow edema of the scaphoid and base of metacarpal 2 and synovitis MCP 4), see Table 5.

**Table 5:** MRI proportion of positive RAMRIS-subscores in male and female patients with hand osteoarthritis (HOA)

Erosions distal radius	HOA female (*n* = 50)	HOA male (*n* = 50)	*p*-value
	6.0%	20.0%	0.038
Erosions base of metacarpal 3	48.0%	18.0%	0.002
BME Scaphoid	6.0%	22.0%	0.022
BME Base of metacarpal 2	0.0%	12.0%	0.012
Synovitis MCP joint 4	66.0%	84.0%	0.039

BME = bone marrow edema, MCP = Metacarpophalangeal

**Table 6:** MRI proportion of positive RAMRIS-subscores in male and female patients with rheumatoid arthritis (RA)

Erosions distal radius	RA female (*n* = 50)	RA male (*n* = 50)	*p*-value
	6.0%	22.0%	0.022
Erosions distal ulna	18.0%	42.0%	0.009
Erosions base of metacarpal 3	62.0%	38.0%	0.017
Erosions base of metacarpal 4	54.0%	32.0%	0.027
Erosions base of metacarpal 5	6.0%	20.0%	0.038

In the distal radius, distal ulnar and the base of metacarpal 5, RAMRIS-subscores of erosions were more prevalent among RA males than RA females, whereas the opposite can be observed regarding the RAMRIS-subscores of erosions at the base of metacarpal 3 and 4 (Table 5).

The female RA were on average more commonly affected than the female HOA, notably in RAMRIS-subscores of erosions of the base of metacarpal 4, MCP proximal 4, MCP distal 5; bone marrow edema of the distal radius, distal ulna, scaphoid, scaphoid, pisiform, hamate and the base of metacarpal 2 and 4.

No differences could be noted amongst HOA smokers and HOA non-smokers and merely the RAMRIS-subscore bone marrow edema of the base of metacarpal 1 was more commonly affected by the inflammatory and degenerative changes amongst RA smokers than amongst HOA smokers. Both RAMRIS-subscores erosions of the base of metacarpal 3 and 4 were more commonly affected amongst RA smokers than amongst HOA smokers, whereas the opposite was true for RAMRIS-subscores of erosions of the trapezium.Joints were more often affected by the inflammatory and degenerative changes amongst RA non-smokers than amongst HOA non-smokers on eight sites (bone marrow edema: distal radius, triquetrum, hamate, trapezoid, base of metacarpal 2, 4 and 5; tenosynovitis: wrist) (Table 7).

**Table 7:** MRI proportion of positive RAMRIS-subscores of non-smoking patients with hand osteoarthritis (HOA) and non-smoking patients with rheumatoid arthritis (RA)

BME distal radius	RA non-smokers (*n* = 50)	HOA non-smokers (*n* = 47)	*p*-value
	12.0%	4.3%	0.003
BME triquetrum	16.0%	8.5%	0.044
BME hamate	16.0%	6.4%	0.039
BME trapezoid	14.0%	8.5%	0.029
BME base of metacarpal 2	10.0%	8.5%	0.037
BME base of metacarpal 4	6.0%	4.3%	0.043
BME base of metacarpal 5	4.0%	2.1%	0.019
Tenosynovitis wrist	72.0%	68.1%	0.010

Additionally, interrater reliability measured by Cohen’s kappa revealed almost perfect agreement between both readers (κ = 0.934; *p* < 0.001).

## Discussion

Our study provides insights into the ability of RAMRIS to differentiate rheumatoid arthritis (RA) from hand osteoarthritis (HOA). The mean scores for RA and HOA were 13.71 vs. 10.92, respectively. As expected, RA patients exhibited significantly higher synovitis subscores, reflecting active inflammation and resulting in overall higher RAMRIS scores [13, 17]. This finding aligns with the established pathophysiology of RA, where persistent synovial inflammation contributes to progressive joint damage [1]. However, a noteworthy observation was that HOA patients also demonstrated synovitis, tenosynovitis, erosions, and bone marrow edema (BME), all features that are traditionally considered hallmarks of inflammatory arthritis, particularly RA [18].

Compared to other studies, we found comparatively low RAMRIS scores in the RA cohort in our study. It is noteworthy that within our cohort, approximately 40% of patients received treatment with cs or bDMARDs, 57% with NSAIDs, and 33% with glucocorticoids, which may provide a rationale for the observed lower scores. Lower RAMRIS scores have also been documented in other active cohorts. Sewerin et al. demonstrated that the Remission-Plus cohort exhibited mean RAMRIS scores of 7.78 at baseline [19], . It should be noted that, due to the heterogeneity of the groups and the different modes of action, no direct comparison between patients is possible in this study. Nevertheless, it should be taken into account that the majority of patients received treatment. In conclusion, the mean RAMRIS scores obtained in this study are consistent with those reported in the extant literature and are indicative of an active RA cohort.

In contrast to rheumatoid arthritis, the RAMRIS has not yet been examined in HOA. In principle, the RAMRIS and its various subscores (synovitis, BME and erosions) were primarily developed for inflammatory diseases and validated for RA [17]. For HOA, which is mainly characterized by osteophytes and bony lesions, conventional radiographic procedures are the gold standard [8]. On the other hand, HOA is very common in the elderly and occurs concomitantly in a high percentage of patients. Giulin et al. showed that 99.8% of a population-based normal cohort had at least 1 osteophyte on ultrasonography and that higher-grade osteophytes occurred significantly more frequently with increasing age [7]. These data showed that osteophytes or other signs of HOA can also be expected in almost all patients in the RA cohort, especially in older age groups. It should also be noted that not all joints primarily affected by HOA are an integral part of RAMRIS. For example, DIP and CMC-1 joints are not assessed. Nevertheless, the evaluation focused on findings in the RA regions that could potentially be misinterpreted as pathological by HOA, rather than on validating the score for typical HOA regions.

The presence of synovitis in HOA suggests that inflammatory changes may be a more common feature in degenerative joint disease than previously assumed. This aligns with prior ultrasound studies that have documented synovial thickening in HOA patients, particularly in cases of so-called activated osteoarthritis [10, 20]. While the degree and distribution of synovitis were more pronounced in RA, our findings underscore that its mere presence should not be misinterpreted as a definitive indicator of RA in MRI assessments. The same applies to tenosynovitis and BME, which were also present in HOA, albeit to a lesser extent. This raises an important diagnostic challenge: while these MRI features are strongly associated with RA, they are not pathognomonic and can also occur in other forms of arthritis, including HOA. The comparatively high RAMRIS scores in the HOA cohort in our studies clearly emphasize that the RAMRIS subdomains primarily classified as inflammatory do not occur exclusively in RA but also in HOA and could thus possibly lead to falsely high scores. This finding should be critically considered when evaluating the MRI.

Erosions, which are a key criterion in RA classification, did not emerge as a highly distinguishing factor in our study. Although erosion scores were slightly higher in RA patients, the difference was not statistically significant. This suggests that erosive changes in HOA can sometimes mimic those observed in RA, especially in later disease stages. Prior studies have similarly reported that osteoarthritic erosions, particularly in the interphalangeal joints, may be difficult to differentiate from those in RA, further emphasizing the importance of integrating imaging findings with clinical and serological data [21].

When considering gender differences, these appear to be particularly relevant for HOA, as men more frequently show synovitis, BME, and erosions in selected joints. This could indicate that particular attention should be given to the interpretation of typical findings, especially in men with concomitant HOA [8, 22]. No relevant differences between the sexes were observed for RA in general. These findings are of particular relevance to everyday clinical practice, as it is well established that HOA occurs more frequently in women than in men. Consequently, a potential bias in the utilisation of imaging procedures, particularly MRI, may be more pronounced in men with concomitant HOA compared to women, in whom HOA may already have been clinically suspected.

Numerous studies have shown that smoking is an important predictor of erosive disease progression. For example, Finckh et al. demonstrated that heavy smokers in particular exhibited significantly more erosive changes than non-smokers after an average of 3.1 years [23]. Looking at the non-smokers in our cohort, BME is significantly more common in RA patients than in non-smoking HOA patients, which further emphasizes the inflammatory nature of the disease.

Despite the above-mentioned insights, some limitations of this study must be acknowledged. First, this was a retrospective study, and the sample size, while considered adequate, was chosen from a single-center cohort which may treat more severe and complex patients than private practices. Considering this, it should be noted that the results may not be fully transferable to other settings. Secondly, we could avoid the usage of intravenous gadolinium imaging as MR contrast agents which have been found to be unfavourable as an imaging technique, as deposits and remnants can be found in the brain [24]. This approach reduces invasiveness and the risk of allergic reactions, enhances patient comfort, and streamlines clinical workflows, leading to improved patient turnaround [16]. However, it should be noted that the RAMRIS score was primarily validated with the use of contrast agents, and deliberately foregoing their use despite good evidence certainly represents a limitation. Moreover, due to data availability matching was only possible for age and sex but not for disease duration or disease stage. This could lead to an imbalance between the groups, which cannot be ruled out with certainty. Finally, it should be noted that the study design was not primarily conceived to distinguish between RA and HOA, and therefore the question of the validity of RAMRIS for assessing HOA does not arise.

In conclusion, our study highlights the utility and limitations of an imaging scoring system in distinguishing lesions in patients with confirmed diagnoses of RA and HOA. We emphasize that MRI findings should always be interpreted qualitatively within the broader clinical context, including patient history, serology, and disease course. While synovitis, BME, and tenosynovitis are more pronounced in RA, their presence alone is not exclusive to the disease. The severity and distribution of synovitis, along with clinical and serological markers, remain key differentiators. Future research should focus on refining imaging-based classification criteria and exploring the role of advanced MRI techniques in improving diagnostic accuracy for inflammatory and degenerative joint diseases.

.

## Data Availability

Due to the sensitive nature of clinical data, datasets are not publicly available but can be obtained from the corresponding author upon reasonable request. P.S and X.B. conceived of the idea. P.S. and J.M.A have compiled the data. All authors interpreted the data. P.S., J.M.A. and XB performed the statistical analyses. X.B. supervised the work. All authors discussed the results and contributed to the final manuscript.
